# Investigating the Relationship Between Self-Perception of Aging and Fear of Falling in Low-Income Community-Dwelling Older Adults

**DOI:** 10.53520/rhm2026.104202

**Published:** 2026-09-22

**Authors:** Carla Stokes Leinbach, Jethro Raphael M. Suarez, Tho Nguyen, Kworweinski Lafontant, Amber Blount, Rui Xie, Nichole Lighthall, Ladda Thiamwong

**Affiliations:** 1 University of Central Florida, Orlando, Florida/USA

**Keywords:** Fall Risk, Fall Prevention, Self-efficacy

## Abstract

**Introduction:**

Fear of falling (FOF) is a key psychological factor associated with fall risk in older adults. Little is known about self-perception of aging (SPA) as a correlate of FOF. This study examined the relationship between FOF and SPA.

**Methods:**

Community-dwelling older adults were recruited from senior programs. SPA was assessed with the Brief-Ageing Perceptions Questionnaire (B-APQ) and FOF with the Short Falls Efficacy Scale-International (Short FES-I). Spearman rank correlation coefficient (ρ) examined associations between FOF and B-APQ subscales.

**Results:**

A total of 286 older adults (mean age = 73.49 ± 6.90 years) were analyzed. Timeline Chronic subscale (ρ = 0.195, p = 0.001) and Emotional Representation (ρ = 0.232, p < 0.001) were positively associated with FOF. Consequences and Control Negative (ρ = −0.295, p < 0.001) and Control Positive (ρ = −0.142, p = 0.018) were negatively associated with FOF. No significant association emerged for Consequences Positive.

**Conclusions:**

Negative SPA dimensions were linked to higher FOF. Among positive dimensions, only Control Positive showed a modest inverse association. Individuals with negative SPA tend to report an elevated FOF and view older adulthood as detrimental, a period of increased loss, and a threat to control. These findings highlight the need to assess internalized aging beliefs. Targeting negative SPA in interventions may reduce FOF by shifting perceptions toward neutral or positive views on aging, informing more effective fall-prevention programs for older adults.

## Introduction

Falls threaten older adults’ health and independence^1^. Fear of falling (FOF), which can be defined as a low perceived self-efficacy in avoiding falls, is a common concern among older adults, as it affects between 20%−85% of the older adult population^2^. Falls and FOF exhibit a cyclic relationship^1^. FOF often leads to a restriction or avoidance of everyday activities,^2,3^ which ultimately fosters frailty through inactivity. This less active lifestyle leads to muscle weakness and balance deterioration, thereby elevating fall risk and perpetuating the loop^2^. Activity restriction, a primary consequence of FOF, affects approximately 40% of community-dwelling older adults^2,3^. Even non-injurious falls trigger self-induced, fear-driven curtailment^2^. The mere threat of falling can induce psychological distress, prompting older adults to limit daily tasks due to FOF^3^. FOF affects both those who have experienced a fall and 65% of those who have not personally experienced a fall^3,4^. FOF can predict future falls but conversely, a history of falls can predict FOF^2^. Additional correlates include advanced age, decreased mobility, chronic illness, being female, knowing someone who experienced a severe fall, institutionalization, and perceived poor health^3,5^.

Beyond physical aging, psychosocial factors contribute to fall risk and FOF^6^. Societal aging stereotypes internalized across the lifespan shape an individual’s self-perception of aging (SPA)^7^. SPA can be defined as the attitudes and feelings that older adults have toward aging and the aging process^7–10^. These perceptions influence their self-concept and overall views of getting older. SPA refers to an individual’s subjective beliefs, attitudes, and expectations about their own aging, encompassing their overall view of aging including their physical, cognitive, and social capabilities as they grow older. Cultural beliefs about aging can shape personal SPA, making some individuals feel less capable as they age. Societal views and frailty-linked stereotypes become part of an individual’s self-perception, can mold SPA, and become part of how older adults see themselves^8^. This process, explained by Stereotype Embodiment Theory^7^, suggests that these views operate unconsciously, intensify with age, embed in self-concept, and alter behaviors such as health-promoting activities like social engagement. For these reasons, SPA can ultimately affect overall health and well-being. SPA can either be positive (aging is associated with wisdom and growth) or negative (aging is associated with decline, loss of control, and dependency) and is increasingly recognized as a key psychological driver of health outcomes and quality of life in older adults^9^.

SPA is a multidimensional construct that is measured and better understood by five subdomains: (1) Timeline-Chronic (TC)- persistent aging awareness, (2) Consequences Positive (ConP)- positive beliefs about the impact of aging, (3) Control Positive (CtrlP)- perceived control over aging, (4) Consequences and Control Negative (CCN)- negative beliefs about the impact of and control over aging, and (5) Emotional Representation (ER)- aging-related negative emotions (anxiety, depression, worry)^10^. Negative SPA frames aging as loss of control, loss of autonomy, and physical decline, while positive SPA views aging as ongoing growth and learning^10^. Although SPA has been associated with multiple health outcomes, relatively little is known about how specific dimensions of SPA relate to FOF. Understanding these relationships may help identify modifiable psychological factors that contribute to FOF and inform interventions that address both physical and psychological determinants of fall risk. Therefore, the purpose of this study was to examine the association between SPA and FOF among community-dwelling low-income older adults. We hypothesized that older adults with more negative SPA, particularly higher scores reflecting losses and lower perceived control over aging, would be associated with greater FOF.

## Methods

### Participants

This cross-sectional study stemmed from a larger grant, federally funded by the National Institute on Minority Health and Health Disparities (R01MD018025) and pre-registered on ClinicalTrials.gov (NCT05778604)^11^. Community-dwelling older adults were recruited in the Orlando, FL area through convenience and snowball recruiting using flyers posted throughout the community and senior social groups. Individuals were eligible for the study if they were at least 60 years of age, were not receiving any form of treatment from a rehabilitation facility, were low-income status based on the 2020 United States Census low-income thresholds^12^, and able to stand unassisted. Individuals were excluded if they were unable to provide informed consent, spoke neither English nor Spanish, were hospitalized more than three times within the 12 months prior to screening, were unable to perform physical activity, or showed signs of cognitive impairment (as determined by a score of 22 or lower on the Rowland Universal Dementia Assessment Scale; RUDAS). The RUDAS is a validated multicultural cognitive screening instrument; a cut-off of ≤22 is the recommended threshold for possible cognitive impairment^13^. Self-reported demographics, such as age, sex, race/ethnicity, and self-rated health, were collected via self-report surveys. The protocol for this study was reviewed and approved by the University of Central Florida Institutional Review Board (STUDY00003206) and carried out in accordance with the Declaration of Helsinki. All participants provided written informed consent prior to participation. All participant data was anonymized, de-identified, and stored per institution-wide policies and HIPAA-compliant policies to ensure privacy within a private Research Electronic Data Capture (REDCap) database.

### Protocol

Participants completed a series of in-person surveys at a singular point in time. SPA was assessed via the 17-item Brief-Ageing Perceptions Questionnaire (B-APQ)^10^, rooted in Leventhal’s Self-Regulation Model of Health and Illness^14^. This model conceptualizes self-perceptions as influencers of coping behaviors^14^. Five subscale domains make up the B-APQ: Timeline Chronic (TC), Consequences Positive (ConP), Emotional Representation (ER), Consequences and Control Negative (CCN), and Control Positive (CtrlP). TC, ER, and CCN reflect negative SPA, while ConP and CtrlP reflect positive SPA^10^. Items are rated on a 5-point Likert scale. TC and ER are scored so that higher values indicate more negative SPA. CCN items are reverse scored so that higher scores reflect more positive SPA. CtrlP and ConP are scored so that higher values indicate more positive SPA. Internal consistency of the study measures was evaluated using Cronbach’s alpha coefficients calculated from the current study sample. The Cronbach’s alpha reliability of each subscale is as follows: TC, α = .655; ConP, α = .799; CtrlP, α = .856; ER, α = .674; CCN, α = .765. FOF was assessed with the validated Short Falls Efficacy Scale-International (Short FES-I)^15^. The instrument includes seven 4-point Likert items assessing the level of concern about falling when engaging in daily living activities (e.g., bathing, going up and down the stairs, getting dressed) and is widely used for determining FOF among older adults. The Short FES-I exhibits excellent internal and test-retest reliability (α=0.92) and is comparable to the FES-I (correlation = 0.97)^15^. The total score ranges from 7 to 28, with scores of 8 or below representing low FOF, scores of 9 to 13 inclusive representing moderate FOF, and scores of 14 or above representing high FOF.

### Statistical Analysis

All statistical analyses were conducted with Statistical Package for the Social Sciences software (SPSS; version 30, IBM, Armonk, NY, USA). Sociodemographic data were summarized descriptively. Categorical variables were presented as frequencies and percentages, while continuous variables were summarized using means and standard deviations. The B-APQ yielded five subscale scores per standard protocol^10^, whereas the Short FES-I score was treated as a continuous variable (higher scores = greater FOF)^15^. Likert composites were treated as interval data. Given potential non-normality, non-parametric Spearman correlations assessed the strength and direction of associations between FOF and each B-APQ subscale. Missing data were not imputed; thus, correlation analyses were conducted using complete observations for each pair of variables. Confidence intervals for coefficients were calculated using the Fisher z-transformation. Statistical tests were two-sided, with statistical significance defined as p < 0.05. No multiple comparison adjustments were made given the exploratory nature of this preliminary study. Correlation coefficients were interpreted based on their magnitude and direction, with confidence intervals reported where appropriate to characterize the precision of the estimates.

## Results

A total of 286 community-dwelling older adults were included in the analysis, of whom 253 (88.5%) were female and 33 (11.5%) were male. Demographic characteristics for all participants are presented in Table 1. Race/ethnicity, education, self-rated health, financial status, living situation, and fall history are likewise reported by sex.

The mean age of participants was 73.49 ± 6.90 years, with a mean BMI of 30.75 ± 6.38 kg/m^2^. The mean Short FES-I score was 10.48 ± 4.20. In this sample, 46.5% had low FOF, 34.3% had moderate FOF, and 19.2% had high FOF. Nearly half of the participants identified as Black (47.9%), followed by Hispanic (24.5%), White (18.5%), Asian (6.6%), and other or undisclosed race/ethnicity (2.5%). Approximately half of the participants (48.6%) had more than a high school education, while 41.6% had completed high school and 9.4% had less than a high school education. Most participants rated their health as good (45.5%) or fair (32.9%), with fewer reporting very good (14.0%), poor (5.6%), or excellent (1.8%) health. Regarding financial status, the majority (59.1%) reported having just enough income to meet their needs, whereas 20.6% reported more than adequate income and 18.9% reported less than adequate income. Over half of the participants (51.8%) lived alone, 24.1% lived with a spouse or partner, and 20.6% lived with family members. Most participants (61.9%) reported no falls in the previous year, while 27.3% experienced one to two falls and 8.0% reported more than two falls. When categorized by sex, male and female participants had similar mean age, BMI, and FES-I scores. More female participants identified as Black (49.8% vs 33.3%) and lived alone (54.2% vs 33.3%). More male participants identified as White (27.3% vs 17.4%) or Asian (15.2% vs 5.5%), had more than a high school education (57.6% vs 47.4%), and lived with a spouse or partner (54.6% vs 20.2%). This distribution of fall history was generally comparable between sexes, although a slightly greater proportion of females reported experiencing more than 2 falls during the previous year (8.7% vs 3.0%).

Correlation analyses were conducted using complete cases for each pair of variables. In Table 2, the TC subscale demonstrated a statistically significant, positive association with FOF (ρ = 0.195, 95% CI: [0.076, 0.309], p = 0.001), indicating that participants who perceived aging-related experiences as more persistent tended to report greater concerns about falling, although the strength of this association was small-to-moderate. Similarly, the ER subscale demonstrated a statistically significant positive association with FOF (ρ = 0.232, 95% CI: [0.114, 0.343], p < 0.001), indicating participants with higher negative aging-related emotions tended to report greater FOF. Although the magnitude of the association was relatively small, the statistically significant correlation suggests that emotional responses and regulation related to aging may be relevant to individuals’ perceptions of fall-related concerns. The CCN subscale (reverse-scored) showed a small-to-moderate negative correlation with FOF (ρ = −0.295, 95% CI: [−0.401, −0.180], p < 0.001). Because higher CCN scores reflect more positive perceptions, this indicates that more positive views on this dimension were linked to lower FOF. The CtrlP subscale was negatively associated with FOF (ρ = −0.142, 95% CI: [−0.259, −0.022], p = 0.018). No statistically significant association was observed between FOF and ConP.

## Discussion

This study examined the relationship between self-perception of aging (SPA) and fear of falling (FOF) among a sample of community-dwelling low-income older adults with no signs of cognitive impairment. It was hypothesized that older adults with negative SPA, particularly greater SPA related to losses and lower perceived control over aging, would report higher levels of FOF than those with positive SPA. This hypothesis was supported by associations between FOF and all three negative SPA subscales. Specifically, results indicated FOF was associated with greater, persistent aging awareness (i.e., Timeline Chronic, TC; ρ = 0.195, p = 0.001), greater aging related negative emotions (i.e., Emotional Representation, ER; ρ = 0.232, p < 0.001), and lower endorsed beliefs about one’s impact of and control over aging (i.e., Consequences and Control Negative, CCN; ρ = −0.295, p < 0.001). Results also revealed a weak inverse relationship with Control Positive (CtrlP; ρ = −0.142, p = 0.018). No significant association was found between FOF and the SPA subscale for positive beliefs about the impact of aging (Consequences Positive, ConP). These findings suggest that, within this preliminary sample, specific negative perceptions of aging may be more closely related to FOF than positive perceptions of aging.

The pattern of associations observed in this sample suggest that negative dimensions of SPA, especially CCN (ρ = −0.295), are more strongly linked to FOF than positive dimensions. CCN captures both anticipated negative consequences of aging (decline, loss of independence) and the belief that the aging process is uncontrollable, indicating these threats can drive greater FOF. In a low-income, predominantly female, racially diverse sample with elevated BMI and mixed health ratings, such loss-and-control beliefs and concerns are especially noticeable. By contrast, ConP was not significantly associated with FOF (Table 2). Beliefs that aging brings wisdom or personal growth are more abstract and less directly relevant to the concrete threat of falling. They may support well-being but do not necessarily alter fall-related self-efficacy. CtrlP was significantly associated with FOF, but this association was modest (Table 2), suggesting that perceived agency over positive aspects of aging buffers some outcomes but does not fully counteract FOF when physical risk factors remain present.

It is well documented that FOF robustly predicts fall risk^1^, but this relationship remains relatively understudied in diverse, low-income groups. In the present study, negative SPA was found to significantly correlate with higher FOF – consistent with prior links to perceptions of poor health^3,5^, negative self-evaluation^7,9,16^, and low self-efficacy^5,7^. Pessimistic views on aging amplify fall-related concerns through psychological and functional pathways^16^. However, positive SPA showed only limited protective association: the CtrlP subscale was modestly related to lower FOF, while the ConP subscale was not significantly associated. This pattern is consistent with a negativity bias, in which adverse views of aging appear to drive fall related concerns, anxiety, and activity restrictions more strongly than positive views mitigate them. This asymmetry aligns with FOF’s ties to physical, psychological, and social declines in older adults^4^. This negative relationship may be influenced by depressive psychological symptoms, poor self-reported health, reduced physical activity^2^, and social withdrawal, thereby leading to the erosion of well-being and increased loneliness. This, in turn, could reinforce fear. Interventions that reframe negative aging beliefs, rather than targeting physical function alone, may interrupt this cycle.^7,17,18^ Existing literature on SPA consistently associates negative SPA with a diminished quality of life, which often involves reduced feelings of psychological well-being and restricted physical and social engagement^17^. This ultimately creates a feedback loop that sustains FOF.

In contrast, positive SPA is linked to enhanced physical health and life satisfaction, potentially offering indirect positive effects which mitigate fall risks by fostering a reinforcing cycle: enhanced SPA boosts self-confidence then encourages social engagement and community-based activity, leading to an increase of physical activity as a natural result of navigating the community. Maintained or improved levels of physical functioning can result from an increase in physical activity. It is well established in the literature that regular physical and social activity boosts perceived health and quality of life^17^. Therefore, improved levels of physical functioning may lead to higher positive SPA and self-confidence while exerting no effect on FOF. This may be because greater physical functioning leads to an absence or decline in serious fall events. This lack of fall events may reduce the chance of FOF development.

Traditional fall risk interventions for older adults have aimed to reduce the risk of falls by reducing FOF through improvements in physical functioning and found mixed results^18,19^. However, our findings support the idea that a reduction of FOF is more nuanced than a singular focus on physiological improvements. The incorporation of brief psychological components such as Cognitive Behavioral Therapy that reframe aging as modifiable rather than inevitable decline could strengthen interventions that already address balance and gait.^7,17,18^ Integrating SPA screenings into routine geriatric care could personalize strategies to reduce FOF and enhance the quality of life for older adults.

Despite this study including a large population sample, numerous limitations exist. This was an exploratory study and therefore utilized a cross-sectional design with correlation analysis to investigate possible associations between variables. The cross-sectional design precludes conclusions regarding causality and therefore, the results should be interpreted with caution. Statistical analyses that control for confounders or a longitudinal study design could clarify FOF-SPA dynamics. Although no adjustment for multiple comparisons was applied given the exploratory nature of the analyses, it should be noted that the association between the CtrlP subscale and fear of falling (p=0.018) would not remain statistically significant under a Bonferroni correction. The reliance upon self-reported data in the form of surveys could have compromised the FOF variable risk via social desirability bias. Additionally, despite recruitment occurring across many senior centers and being open to any individual within those centers, the study sample size exhibited an overrepresentation of Black participants (47.90%) and an underrepresentation of males (11.54%). Additionally, the study was conducted in a single geographic location. Such characteristics may limit the generalizability of the results. Future work should consider measuring SPA and FOF across multiple time points and performing statistical analyses that control for possible confounders, emphasizing to participants that all responses to the surveys are confidential and will not be shared with anyone to reduce possible social desirability bias, and improving the generalizability of the findings by recruiting a more diverse sample population with a more balanced representation of racial/ethnic groups and sexes.

## Conclusions

The results from this study show that negative SPA exhibited statistically significant, small-to-moderate correlations with FOF among a community-dwelling low-income older adult population. These findings show that FOF potentially extends beyond physical vulnerability^2^. Negative SPA is characterized as viewing older adulthood as a period of increased risk for loss, as well as a threat to control, and is a factor that psychologically erodes an individual’s perceived fall-avoidance efficacy. Although multifaceted, FOF may be influenced by modifying an older adult’s beliefs and concerns about aging. Overall, the findings are consistent with negative bias: negative aging beliefs are more strongly associated with FOF than positive beliefs protect against it. This supports interventions that specifically reframe negative consequence-and-control perceptions. Through psychological interventions such as cognitive reframing, SPA may be used as an additional pathway to modify the physiological threat of falling. These study findings broaden understanding of FOF from a less-widely considered psychological lens.

## Figures and Tables

**Table 1.** Demographic characteristics.

Characteristic	Total (N=286)	Male (n=33)	Female (n=253)
Age (years)	73.49 ± 6.90	72.64 ± 6.87	73.60 ± 6.91
BMI (kg/m^2^)	30.75 ± 6.38	30.83 ± 6.47	30.74 ± 6.39
B-APQ Score	56.24 ± 5.14	56.28 ± 5.18	56.23 ± 5.15
FES-I Score	10.48 ± 4.20	10.03 ± 4.41	10.53 ± 4.18
Race/Ethnicity			
Asian	19 (6.64%)	5 (15.15%)	14 (5.53%)
Black	137 (47.90%)	11 (33.33%)	126 (49.80%)
Hispanic	70 (24.48%)	8 (24.24%)	62 (24.51%)
White	53 (18.53%)	9 (27.27%)	44 (17.39%)
Other/Did not disclose	7 (2.45%)	0 (0.00%)	7 (2.77%)
Education Level			
Less than high school	27 (9.44%)	1 (3.03%)	26 (10.28%)
High school	119 (41.61%)	13 (39.39%)	106 (41.90%)
More than high school	139 (48.60%)	19 (57.58%)	120 (47.43%)
Did not disclose	1 (0.35%)	0 (0.00%)	1 (0.40%)
Self-rated Health			
Poor	16 (5.59%)	3 (9.09%)	13 (5.14%)
Fair	94 (32.87%)	13 (39.39%)	81 (32.02%)
Good	130 (45.45%)	12 (36.36%)	118 (46.64%)
Very Good	40 (13.99%)	5 (15.15%)	35 (13.83%)
Excellent	5 (1.75%)	0 (0.00%)	5 (1.98%)
Did not disclose	1 (0.35%)	0 (0.00%)	1 (0.40%)
Financial Status			
Less than adequate	54 (18.88%)	8 (24.24%)	46 (18.18%)
Just enough	169 (59.09%)	17 (51.52%)	152 (60.08%)
More than adequate	59 (20.63%)	8 (24.24%)	51 (20.16%)
Did not disclose	4 (1.40%)	0 (0.00%)	4 (1.58%)
Living Situation			
Alone	148 (51.75%)	11 (33.33%)	137 (54.15%)
With spouse/partner	69 (24.13%)	18 (54.55%)	51 (20.16%)
With family	59 (20.63%)	4 (12.12%)	55 (21.74%)
Other/ Did not disclose	10 (3.50%)	0 (0.00%)	10 (3.95%)
History of Falls			
No fall	177 (61.89%)	23 (69.70%)	154 (60.87%)
1–2 falls	78 (27.27%)	9 (27.27%)	69 (27.27%)
More than 2 falls	23 (8.04%)	1 (3.03%)	22 (8.70%)
Did not disclose	8 (2.80%)	0 (0.00%)	8 (3.16%)

Data are presented as mean ± standard deviation (SD) for continuous variables and frequency (percentage) for categorical variables.

**Table 2.** Spearman’s Rho Correlation (ρ) between B-APQ domains and FOF (Short FES-I).

B-APQ Domain	n	ρ	95% CI	p-value
**TC**	278	0.195	0.076, 0.309	0.001
**ER**	278	0.232	0.114, 0.343	<0.001
**CCN**	278	−0.295	−0.401, −0.180	<0.001
**ConP**	278	−0.106	-0.224, 0.015	0.077
**CtrlP**	278	−0.142	−0.259, −0.022	0.018

Correlation analyses were conducted using complete cases for each pair of variables. Higher CCN scores reflect more positive perceptions of aging.
